# Study of the upper pole after subtotal splenectomy in rats

**DOI:** 10.1590/acb371103

**Published:** 2023-01-06

**Authors:** Amanda Lessa Martins, Anna Bárbara Scárdua Parreira, Maria Luiza Font Juliá Grossi, Raquel de Azevedo Benevides, Luciene Lage da Motta, Lucia Helena Sagrillo Pimassoni, Andrea Saade Daher Borjaili, Marcela Souza Lima Paulo, Danilo Nagib Salomão Paulo

**Affiliations:** 1 Graduate student. Escola Superior de Ciências da Santa Casa de Misericórdia de Vitória – School of Medicine – Vitória (ES), Brazil.; 2 Veterinarian. Autonomous – Vitória (ES), Brazil.; 3 PhD, full professor. Escola Superior de Ciências da Santa Casa de Misericórdia de Vitória – Department of Pathology – School of Medicine – Vitória (ES), Brazil.; 4 PhD, Full Professor. Escola Superior de Ciências da Santa Casa de Misericórdia de Vitória – Department of Statistics – School of Medicine – Vitória (ES), Brazil.; 5 PhD. State Children’s Hospital Nossa Senhora da Glória – Pediatric Surgery – Vitória (ES), Brazil.; 6 PhD, associate professor. Escola Superior de Ciências da Santa Casa de Misericórdia de Vitória – School of Medicine – Vitória (ES), Brazil.; 7 PhD, full professor. Escola Superior de Ciências da Santa Casa de Misericórdia de Vitória – Department of Surgery – School of Medicine – Vitória (ES), Brazil.

**Keywords:** Spleen, Splenectomy, Rats

## Abstract

**Purpose:**

To evaluate macro/microscopic viability of the upper pole (UP) in rats after 80 days of subtotal splenectomy preserving the upper pole (SSPUP).

**Methods:**

Twenty-five male Wistar rats were submitted to SSPUP. After 80 days, the rats were euthanized, and the remaining UP was evaluated macroscopically regarding appearance, color, consistency, length, width, thickness, and presence of fibrosis/necrosis; and microscopically regarding presence of red and white pulp, fibrosis/necrosis.

**Results:**

Two rats died during surgery and were removed from the statistical analysis. There was statistically significant increase in length and width between the pre and postoperative in the experimental group, with no significant difference in thickness. In the manipulation group, the macroscopic appearance of the spleen was normal in pre and postoperative, with viability preserved. In the experimental group, two UP of the spleen were not found during the second surgery. Macroscopically, it was observed absence of fibrosis and necrosis in all cases. Microscopically, the white and red pulp were intact in both groups. Two spleens of rats in the manipulation group presented areas with fibrosis and necrosis focus, which were not enough to be considered inviable.

**Conclusions:**

The UP of the spleen remained viable in 91.3% of the cases.

## Introduction

For a long time, spleen functions were unknown, and the importance of this organ was little explained. Therefore, its absence was considered compatible with life^1^. The belief was that its removal due to traumas or other diseases would not cause further damages to the patient^1^. Thus, total splenectomy was always recommended, with no scientific criteria^1^^,^ 2.

Splenectomy registered postoperative deaths for both sexes. Data collected from the Informatics Department of Brazilian Public Health System (DATASUS) point out 36 deaths registered, 24 males and 12 females, in the state of Espírito Santo, Brazil, from 2005 to 2019 in the category Internacional Classification of Diseases 10 (ICD-10): A40 Streptococcal sepsis, D73 Diseases of spleen. In the period from January 2015 to December 2020, the mortality rate for individuals that were submitted to total splenectomy in Brazil was 5.36, with the number of deaths for this same period equal to 906^3^.

The spleen is the largest secondary lymphoid organ, and it has a key role in body’s defense through phagocytosis and filtration mechanisms, besides the production of complement factors and immunoglobulins. Furthermore, this organ can filter pathogens and abnormal cells in the blood, and it has an important participation in hematopoiesis and depuration of red blood cells^2^^,^ 4.

In 1919, with the studies of Morris and Bullock^5^, it was recognized the relation between total splenectomy and increased susceptibility to infection, since these individuals would be less able to control the condition of sepsis. King and Schumacker Jr^6^, in 1952, when describing post-splenectomy fulminant sepsis, raised the attention of the scientific community towards the importance of the splenic tissue in the prevention of severe infections. The risks of post-splenectomy infection, mainly in the first years after the surgery, have been observed in children and adults^7^^,^ 8. In addition, post-splenectomy infectious complications have already been observed in experimental animals^5^^,^ 8. Changes in the lipid metabolism have already been observed in human beings^9^^,^ 10 and laboratory animals^11^, which can cause atherosclerosis. In 1962, Campos^12^ presented partial splenectomy in the surgical practice with the proposal of preserving enough splenic tissue for the body to maintain its defense function.

The spleen is an organ that holds great importance. Therefore, its total or partial preservation began to be more frequently performed through splenorrhaphy^1^, vascular occlusion^13^^,^ 14, partial splenectomy^15^^,^ 16 and splenic autotransplantation^17^^,^ 18. Since 1984, in the attempt to maintain at least part of the splenic function when spleen removal is recommended, it was performed subtotal splenectomy preserving the upper pole (SSPUP), which is nourished by splenograstric vessels^19^. Further studies in this area revealed the possibility of performing this surgical procedure laparoscopically^2^. In his studies, the researcher Petroianu^2^ shows this procedure as a new way of treating severe pain caused by spleen ischemia, splenic trauma, myelofibrosis and myeloid metaplasia, cystadenoma in the pancreatic tail, Gaucher disease, and, at last, for the treatment of hemorrhage from intestinal varices, due to portal hypertension^20^.

Paulo’s most recent study^21^, conducted in 2020, compared the viability of the upper pole (UP) and lower pole of the spleen 80 days after partial splenectomy, which did not present significant statistical difference and were both viable. Although the study monitored animals for an extended period of time, there were no studies that monitored animals after SSPUP surgery for a similar period.

After performing SSPUP, it is believed that the immune function of the spleen would not cease, decreasing the occurrence of immunodeficiencies and, consequently, generalized infection. As previously mentioned, so far, there are no studies monitoring animals for an extended period of time, which would be necessary to effectively evaluate the validity of UP after SSPUP.

Thus, the aim of this study were to verify if the UP is able to remain viable after removal of the middle and lower portion of the splenic tissue, as well as comparing UP size for the experimental group on the first day and 80 days after SSPUP; comparing macroscopic appearance of UP for the experimental group on the first day and 80 days after the surgical procedure; comparing macroscopic appearance of the spleen for the experimental group and manipulation group 80 days after the first surgical procedure; and to evaluate microscopic appearance of UP for the experimental group 80 days after the surgical procedure to compare with the manipulation group.

## Methods

This study was conducted in the Animal Experimentation Bioterium in the Research Center of the Escola Superior de Ciências da Santa Casa de Misericórdia de Vitória, after approval of the Ethics Committee on the Use of Animals, under protocol number 004/2018. Animal manipulation followed the recommendations of the National Council for the Control of Animal Experimentation.

### Sample characteristics and animal care

Twenty-five male Wistar rats (*Rattus norvegicus*) were used, 2 months old, weighing 314.07 g ± 25.62, from the Bioterium of Universidade Vila Velha, properly identified and kept in acclimatized environment inside appropriate cages in the Bioterium Cabinet STD 5 (Grupo Vidy, São Paulo, Brasil), with control of temperature (20 to 22 °C), ventilation and light (12 hours of light and 12 hours of dark). They were fed with food for laboratory rats and water *ad libitum* in all stages of the experiment. The animals were randomly distributed through sorting, in two groups:

- Manipulation group (n = 8): spleen manipulation;
- Experimental group (n = 17): SSPUP.

The rats were allocated in groups of two or three in each cage, identified and monitored individually during the entire experiment.

### Surgical procedure

The animals were weighed (electronic balance Filizola^®^ model MF-6) and anesthetized with ketamine hydrochloride 75 mg/kg(Vetaset^®^, Fort Dodge-Iowa, United States of America) combined with Xylasina hydrochloride 5 mg/kg (Kensol^®^, König-Avellaneda, Argentina) injected intraperitoneally. Next, they were immobilized in the operating table, where trichotomy and antisepsis of the abdominal wall were performed with a 2% iodized alcohol solution and placement of a fenestrated surgical field.

**Figure 1:**
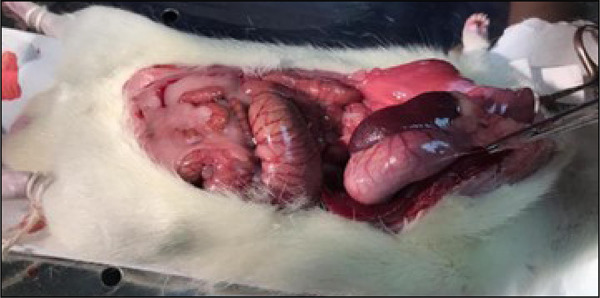
Median laparotomy for examinationof the abdominal cavity.

In the sequence, a median laparotomy with about 2.5 cm long was performed, starting 0.5 cm below the xiphoid process and examination of the abdominal cavity (Fig. 1).

**Figure 2:**
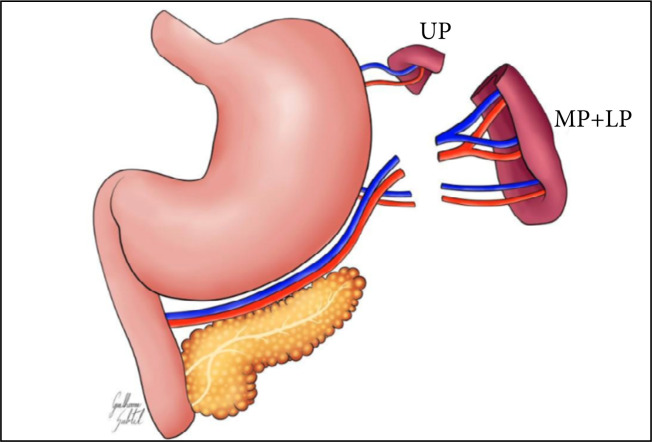
Schematic drawing of the surgeryperformed in this study.

UP: upper pole maintained; LP: lower pole removed; MP: middle portion removed.

In the manipulation group, only the manipulation of the spleen was performed. In the experimental group, the middle and lower portion of the spleen were removed along with their supply vessels, maintaining the UP of the organ according to the techniques recommended by Petroianu^22^ (Fig. 2).

### Description of SSPUP

- After immobilization of the spleen in the surface of the abdominal cavity, the splenic vessels that irrigate the middle and lower portion of the spleen were connected and sectioned, while the possible vessels destined to the UP were maintained;
- The spleen was sectioned with removal of its middle and lower portions, which were placed in a bottle with 10% formaldehyde;
- The UP of the spleen was maintained and measured (length, width, and thickness) in its central part, with a manual pachymeter^23^;
- After performing the SSPUP, abdominal wall closure was performed through continuous sutures of the peritoneum and the muscle-aponeurotic plan together. Then, the skin was sutured with mononylon 6 (Shalon^®^, Shalon Fios Cirúrgicos LTDA, Goiás, Brazil).

In the immediate postoperative period, the animals received dipyrone sodium (Medley, São Paulo, Brazil) at the dose of 52.5 mg/day, orally, dissolved in drinking water (72 hours), free diet and free water. It was also used nalbuphine hydrochloride (Nubain^®^, Cristália, São Paulo, Brazil) at the dose of 0.1 mg/kg of weight applied to the subcutaneous, every 12 hours, for three days. The evolution of animals was registered in individual files.

### Collection of the upper pole and euthanasia of animals

On the 80th postoperative day, the animals were submitted to a second surgery, in which they were anesthetized again and submitted to a new laparotomy. The UP of the spleen was measured (length, width, and thickness), the same was performed during SSPUP, and macroscopically evaluated. Then, the remaining tissue was placed in a bottle with 10% formaldehyde for further microscopic analysis. At last, euthanasia was performed with sodium pentobarbital 120 mg/kg intraperitoneally and potassium chloride 10% (dose-effect), with intracardiac injection.

### Macroscopic evaluation

During exploration of the abdominal cavity of the rats, appearance, color, consistency, size, and presence or not of fibrosis and necrosis in the UP of the spleen were verified.

### Microscopic evaluation of the spleen and its poles

In the microscopic evaluation, the middle and lower portion of the spleen of the experimental group were placed in a solution with 10% formaldehyde on the day of the first surgery, while the UP of the experimental group and the spleen of the manipulation group were removed on the 80th postoperative day, included in paraffin blocks and cut in a rotating microtome with 3 μm thick. Histological sections were placed on glass slides and in an oven at 58 °C for 24 hours. Adhered to the slides, these cuts were dewaxed in xylol and stained using Masson’s hematoxylin-eosin (H&E) and trichrome methods. Microscopy was performed by a pathologist using a binocular microscope. The cuts were analyzed for the following parameters: presence of red pulp, presence of white pulp, fibrosis, necrosis, and viability.

### Studied variables and statistical tests

The categorical variables–appearance, color, consistency, fibrosis, necrosis, and viability–were organized through frequencies and percentages, while the quantitative ones–length, width, and thickness–were summarized using measures such as mean, median, standard deviation, minimum, and maximum. The associations between qualitative variables were performed by the χ^2^ test, while the comparisons were performed using Wilcoxon’s non-parametric test, once the variables did not present normal distribution by the Kolmogorov-Smirnov’s test (p > 0.05). Binomial test was used to compare appearance, color, and consistency of the UP on the pre and postoperative for the experimental group. Associations and comparisons were considered significant when p < 0.05. The data was tabulated in Excel sheet and analyzed using the IBM program Statistical Package for the Social Sciences (SPSS) Statistics version 27 and Bioestat 5.3.

## Results

Among the 25 rats used in the research, there was a survival rate of 92%. Two animals from the experimental group died during the first surgery and were removed from the study. Then, the analysis was done with eight rats from the manipulation group and 15 rats from the experimental group.

### Measures of the upper pole

On Table 1, there is a comparison of length, width, and thickness of UP in the preoperative and postoperative for the experimental group. UP increased in length, width, and thickness from the beginning to the end of the experiment. However, the result points out significant difference between pre and post (in the experimental group) for length and width variables (p < 0.05).

**Table 1:** Length, width and thickness of upper pole in the preoperative and postoperativefrom the experimental group (n = 15)#.

Characteristic	Mean	Standard deviation	Median	Minimum	Maximum	p-value
Length						
Preoperative (mm)	7.9	1.8	8	5	11.5	0.002*
Postoperative (mm)	11.8	1.7	11	10	16	
Width						
Preoperative (mm)	6.3	1.5	6	4	10	0.022*
Postoperative (mm)	7.8	1.1	8	6	10	
Thickness						
Preoperative (mm)	4.1	0.9	4	2.5	6	0.057
Postoperative (mm)	5.1	1.4	5	3	8	

# Wilcoxon’s Test;

p-value: comparison of length, width, and thickness of upper pole in the preoperative and postoperative for the experimental group, considering

* p < 0.05 significant and p > 0.05 non-significant.

### Macroscopic evaluation of the upper pole

In the manipulation group, the macroscopic appearance of the spleen was normal, with brown color, elastic consistency, and absence of fibrosis and necrosis, both in the pre and postoperative, with viability preserved. In the experimental group, in turn, it was observed that most UP were macroscopically viable, with only two (13.33%) considered inviable in the postoperative, since they were not found during the second surgery. Therefore, it was observed no statistical significance in the comparison between macroscopy in the pre and postoperative for the experimental group. This shows that the proportion of normal appearance, brown color, and elastic consistency in the preoperative was similar to the postoperative (Table 2).

**Table 2:** Appearance, color and consistency of UP in the preoperative and postoperative from the experimental group (n = 15)#.

Appearance	Normal	UP absent	p-value
Preoperative	*15*	*0*	*0.1432*
Postoperative	13	2	
**Color**	**Brown**	**UP absent**	**p-value**
Preoperative	*15*	0	*0.1432*
Postoperative	13	2	
**Consistency**	**Elastic**	**UP absent**	**p-value**
Preoperative	*15*	0	*0.1432*
Postoperative	13	2	

UP: upper pole;

# binomial test to compare two proportions; p-value: comparison of appearance, color, and consistency of UP in the preoperative and postoperative for the experimental group, considering *p < 0.05 significant and p > 0.05 non-significant.

In the comparison of macroscopic results between experimental group and manipulation group, it was verified no association of appearance, color, and consistency between the groups (experimental and manipulation) (Table 3).

**Table 3:** Appearance, color, and consistency of upper pole in the postoperative in the experimentalgroup and in the manipulation group#.

	Experimental (n = 15)	Manipulation (n = 8)	p-value
Appearance			0.761
Normal	13	8	
Absent	2	0	
**Color**			**0.761**
Brown	13	8	
Absent	2	0	
**Consistency**			**0.761**
Elastic	13	8	
Absent	2	0	

# χ^2^ test; p-value: comparison of appearance, color, and consistency of upper pole in the postoperative between experimental group and manipulation group, considering *p < 0.05 significant and p > 0.05 non-significant.

**Figure 3:**
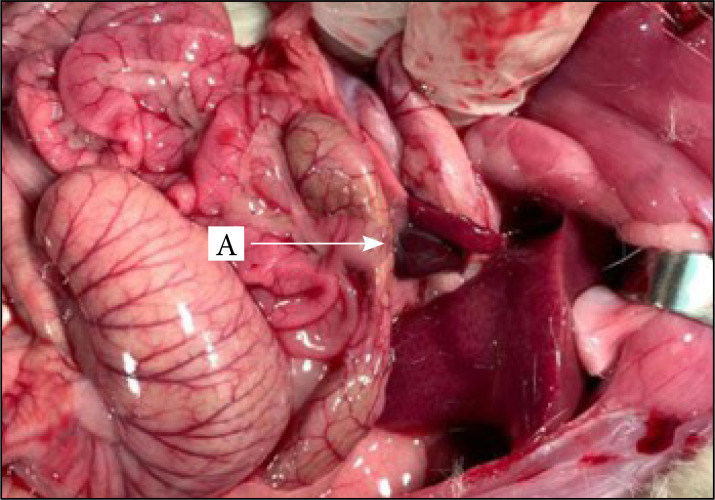
Immediate result after the removal of the middle and lower portion of the spleen in rats ,with preservation of the UP.

**Figure 4:**
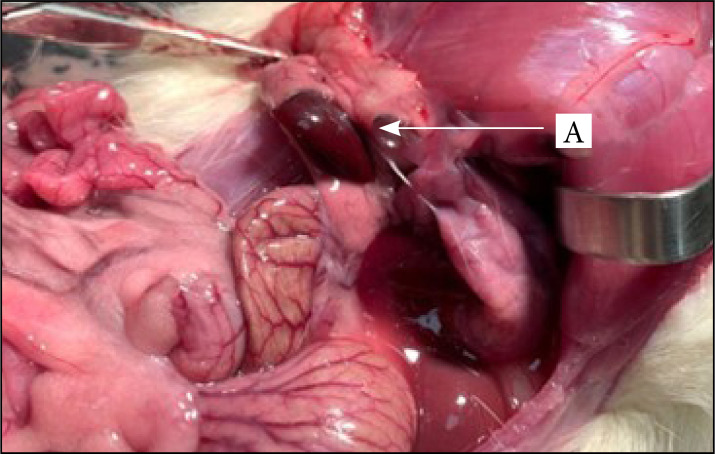
Late result, 80 days after the removal of the middle and lower portion of the spleen, with preservation of UP.

Figures 3 and 4 show and exemplify, respectively, the immediate and late results 80 days after the removal of the middle and lower portion of the spleen, from the surgical procedure of the experimental group.

A: Upper pole.

A: Upper pole.

When analyzing the presence of fibrosis and necrosis in the UP of experimental group, it was observed in the preoperative the absence of both processes in all cases. In the postoperative, in turn, among the UP found (86.7%), it was verified absence of fibrosis and necrosis. Regarding the manipulation group, the result was similar to the one found for the preoperative of experimental group, that is, 100% of spleens did not present fibrosis and necrosis.

### Microscopic evaluation of the upper pole

Most of the UP of animals in the manipulation group and experimental group were viable. The white pulp and red pulp were intact in rats from manipulation group and experimental group. In the manipulation group, only two spleens of rats (13.33%) presented fibrosis, and only two UP of rats in the experimental group (13.33%) presented necrosis, which was not enough to consider them enviable. It was observed that only two (13.33%) of the UPs were enviable once they were not found during the second surgery. In the combination of findings between the groups, the absent UP were not included in the statistical analysis, because it was not possible to evaluate them. There was no significant association between the presence or not of fibrosis or necrosis between experimental and manipulation groups (Tables 4 and 5).

**Table 4:** Presence of fibrosis in the postoperative in the experimental group and in the manipulation group#.

		Experimental (n = 15)			Manipulation (n = 8)	
		Counting	%		Counting	%
Fibrosis	Negative	13	86.7		7	87.5
	Positive	0	0		1	12.5
	Upper pole absent	2	13.3		0	0

# χ^2^ test; p-value: association of fibrosis in the postoperative between experimental and manipulation groups, considering *p < 0.05 significant and p > 0.05 non-significant.

**Table 5:** Presence of necrosis in the postoperative in the experimental group and in the manipulation group#.

		Experimental (n = 15)			Manipulation (n = 8)	
		Counting	%		Counting	%
Necrosis	Negative	12	80		8	100
	Positive	1	6.7		0	0
	UP absent	2	13.3		0	0

# χ^2^ test; p-value: association of necrosis in the postoperative between experimental and manipulation groups, considering *p < 0.05 significant and p > 0.05 non-significant.

## Discussion

With advances in technology and a better understanding around the splenic vasculature, it was observed that total splenectomy could be avoided, and new ways of intervention could be established. This could also guarantee the treatment of splenic complications and the organ to remain functioning in the body^24^^,^ 25. This study had the aim of proving the viability of the UP of the spleen of rats after performing SSPUP. The expected result was for most UP of rats that underwent surgery not to have structural or functional changes, as well as the rat’s health condition, which should evolve with no abnormalities.

Among the 25 rats, there was a survival rate of 92%. Two animals from the experimental group died during the first surgery and were removed from the study. The reason for this occurrence is believed to be an anesthetic overdose that happened during induction. Nevertheless, all animals that underwent SSPUP survived the surgical procedure after 80 days, which suggests a successful surgery. The period of 80 days was estimated considering Paulo’s study on the growth of the lower pole (LP) of the spleen after subtotal splenectomy with preservation of the lower pole (SSPLP) in rats, in which the variables were compared on the 80th postoperative day and there were promising results. In the study, the remaining of the spleen presented significant growth and compatible signs with cell hyperplasia using optical microscopy with light^26^.

A study was conducted in 2020^21^ in which the surgical techniques of Paulo *et al*.^16^ and Petroianu^22^ were performed for the removal of the middle portion of the spleen and its supply vessels, maintaining the UP and the LP of the organ. The study did not present a significant change for the LP regarding the parameters of length, width, and thickness, while there was only significant growth for the UP length. One hypothesis suggested by Paulo *et al*. was that the maintenance of two poles together could have suppressed the growth of both. In another study by Paulo *et al*.^26^, the length, width, and thickness of the LP of rats increased in 68.4% of the animals that underwent SSPLP between the first and the 80th postoperative day. In this study, when comparing length, width, and thickness in the experimental group, it was observed significant growth between the pre and postoperative for the parameters of length and width, which indicates the viability of the organ after SSPUP. On the other hand, thickness presented growth, but it was not considered significant. This suggests that the presence of both poles could have affected the growth of one and the other.

However, it should be considered that the growth of the remaining spleen could be due to the rat’s growth, an inflammatory or traumatic process, the compensatory function of the spleen compromised by the removal of one of its parts, or the combination of all the factors^26^. Regarding inflammation, in this study there were no signs of inflammation enough to explain the phenomenon.

In the macroscopic analysis, 91.3% of the rats presented the UP of the spleen with normal appearance, brown color, elastic consistency, and absence of fibrosis and necrosis, which indicates that they were all viable. However, the UP of the spleen was not found in two rats from the experimental group, which indicates autolysis of this segment. This could be associated to insufficient irrigation provided by splenogastric vessels, which was not able to nourish the UP. Another hypothesis would be the section of the gastrosplenic peritoneal membrane, which is responsible for joining the spleen to the stomach preventing the rotation of the splenic pedicle. This section could have caused torsion in the splenograstric vessels, resulting in ischemia of the remaining part of the organ and, consequently, the resorption of it. However, this fact cannot be affirmed regarding its occurrence or not. Mendonça *et al*.^27^ reported that section of the gastrosplenic peritoneal membrane in rats submitted to SSPLP did not change the viability of the LP. A further study with a group of rats using section and other one with no section of the gastroplenic membrane would better clarify the effect of preserving the membrane in the viability of the UP. Verifying the influence of section, the membrane associated with viability of the remaining UP would be interesting to analyze possible causes of the autolysis identified in our study.

Considering the growth in the organ in most cases (length grew in 100% of cases; width in 66.7%; and thickness in 40%) and the macroscopic results obtained, it was possible to conclude that it is viable to perform SSPUP with the intention of preserving the immune function of the spleen, avoiding the occurrence of immunodeficiencies and generalized infection. The findings in this study reinforce the credibility and safety of performing the surgery in the medicinal field, substituting the primitive way of surgical intervention. Thus, several disorders that exist in the postoperative of total splenectomy can be avoided^24^^,^ 25. However, it should be considered that this study was conducted on rats, and its applicability for human beings should be performed with prudence.

Borjaili *et al*.^28^ compared the survival rates of rats submitted to SSPLP, total splenectomy, and spleen manipulation after induction of fecal peritonitis. The results showed that maintenance of the splenic tissue through SSPLP helped to increase the survival time of rats^28^. This study reinforces the functionality of the conservative surgery with preservation of the LP, but further studies are necessary with the aim of proving that SSPUP is also able to guarantee increased survival rate in rats after inducing fecal peritonitis.

Microscopic viability of the spleen was proven through the presence of white pulp and red pulp, characteristics of the organ. Only one spleen of a rat from the manipulation group presented fibrosis, and one spleen of a rat from the experimental group presented necrosis, which can be related to a possible irrigation deficit in the UP of the spleen. However, the presence of these areas was considered with no representation and insufficient to consider the UP of the spleen inviable. The results corroborate Paulo *et al*.^27^, whose findings in the microscopy confirmed the viability of both poles, with the presence of white pulp (lymphoid follicles, and their components), red pulp, vascularity, and cellularity specific to the organ, in addition to possible inflammatory processes.

Taking into account the results presented, it can be considered that maintenance of the UP through performance of SSPUP would allow continuity of the immune function of the spleen, decreasing the occurrence of immunodeficiencies and, consequently, generalized infection and mortality rates regarding individuals that are submitted to total splenectomy^24^.

## Conclusion

Considering that the UP of the spleen grew significantly in a period of 80 days, and its macro and microappearance are similar to the one of the manipulation group, it can be affirmed that the pole mentioned maintained its viability, which occurred in 91.3% of cases.
